# Kindlin 2 Regulates Myogenic Related Factor Myogenin *via* a Canonical Wnt Signaling in Myogenic Differentiation

**DOI:** 10.1371/journal.pone.0063490

**Published:** 2013-05-22

**Authors:** Yu Yu, Lihua Qi, Junzhou Wu, Yunling Wang, Weigang Fang, Hongquan Zhang

**Affiliations:** 1 Key Laboratory of Carcinogenesis and Translational Research, Ministry of Education of China, Peking University Health Science Center, Beijing, China; 2 Laboratory of Molecular Cell Biology and Tumor Biology, Department of Anatomy, Histology and Embryology, Peking University Health Science Center, Beijing, China; 3 Department of Pathology, Peking University Health Science Center, Beijing, China; The University of Hong Kong, Hong Kong

## Abstract

Kindlin 2, as an integrin-associated protein, is required for myocyte elongation and fusion. However, the association of Kindlin 2 with muscle differentiation-related signaling pathways is unknown. Here, we identified a mechanism that Kindlin 2 regulates myogenic regulatory factors myogenin via a canonical Wnt/β-catenin signaling. We found that knockdown of Kindlin 2 leads to the abolishment of β-catenin/TCF4-mediated transcription in C2C12 cells, followed by the impairment of myogenic differentiation. Mechanistically, nuclear translocation of both Kindlin 2 and β-catenin is induced during myogenic differentiation. In particular, Kindlin 2 forms a tripartite complex with active β-catenin and TCF4, and hence co-occupied on the promoter of myogenin to enhance its expression. Functionally, depletion of Kindlin 2 impairs myogenic differentiation via downregulation of myogenin. Taken together, our data reveal that Kindlin 2 is required for Wnt signaling-regulated myogenic differentiation, providing a mechanistic insight into the role of Kindlin-2 in muscle development.

## Introduction

Skeletal myogenesis is a complex and highly regulated process that mediates the formation of multinucleated myotubes by fusion of mononucleated myoblasts. During myogenesis, Wnt signaling plays an essential role [1]. Activation of Wnt/β-catenin signaling leads to the accumulation of β-catenin, which enters into nucleus and forms a complex with T-cell factor/lymphoid enhancer factor (TCF/LEF), activating the expression of myogenic regulatory factors (MRFs) [2]–[4]. MRFs, a family of myogenic basic helix-loop-helix transcription factors, include myogenin, Myf5, MyoD, and MRF4 which are essential for myogenesis and muscle development [1], [5], [6]. Dysregulation of Wnt pathway leads to aberrant muscle differentiation and muscle development [7].

Integrins and proteins that bind to integrins are required for muscle development and muscle function [8]. Fermitin family homolog 2 (FERMT2, Kindlin 2) is a member of structurally similar and evolutionarily conversed fermitin family. Kindlin 2 is well characterized in regulating integrin activation and mediating cell-cell or cell-matrix adhesion [9], [10]. Kindlin 2, as an integrin-binding partner, plays a critical role in muscle development. Kindlin 2 is highly expressed in cardiac muscle [11], and is required for myocardial formation and function [12]. Moreover, Kindlin 2 is also involved in the skeletal development [13]. Dowling et al. identified that Kindlin 2 regulates myocyte elongation and is essential for myogenesis [13]. Taken together, the role of Kindlin 2 in vertebrate muscle development is definite, however, how Kindlin 2 functions remains little known.

Our recent study indicated that Kindlin 2 is a novel regulator of Wnt signaling. The Kindlin 2 in the cytoplasm is mainly responsible for stabilizing β-catenin, whereas the Kindlin 2 in the nucleus significantly activates the target genes of Wnt signaling [14]. Given the essential role of Wnt signaling in muscle development, we hypothesize whether Wnt signaling mediates the regulation of Kindlin 2 on myogenesis. In our study, Kindlin 2 is accumulated in the nucleus of differentiated C2C12 cells and forms a tripartite complex with β-catenin and TCF4. Moreover, the tripartite complex co-occupies on the promoter of myogenic regulatory factor myogenin, activating the expression of myogenin and inducing myogenic differentiation.

## Materials and Methods

### Cell Culture

C2C12 cells were grown in Dulbecco’s modified Eagle’s medium (DMEM; Invitrogen) supplemented with 15% fetal bovine serum (FBS, Invitrogen), and an appropriate amount of penicillin/streptomycin in a 37°C, 5% CO_2_ humidified incubator. To induce myogenic differentiation, the growth medium (GM) containing 15% FBS was substituted with differentiation medium (DM) contained 2% horse serum. The differentiation medium was exchanged every day.

### Western Blotting

Both Western blotting and Co-IP assay were performed as described previously [14]. The antibodies used were anti-Myosin Heavy Chain (MyHC, Millipore, clone A4.1025), anti-β-catenin (Santa Cruz Biotechnology), anti-Kindlin 2 (Millipore), anti-active β-catenin (Millipore 8E7), anti-TCF4 (Millipore), anti-myogenin (epitomics).

### Real-time PCR (qPCR)

Total RNA was extracted using Trizol reagent (Invitrogen). cDNA was synthesized using the SuperScript kit (Invitrogen). The primer sequences were as followed: Kindlin 2 forward primer, 5′- AGTGGAATGTCAACTGGGAGATC -3′ and reverse primer, 5′- GGACAACCGGACCTCATCTG-3′; MyHC forward primer, 5′- AGAAGGAGGAGGCAACTTCTG-3′ and reverse primer, 5′- ACATACTCATT GCCGACCTTG-3′; GAPDH forward primer, 5′- TGTGTCCGTCGTGGATCTGA -3′ and reverse primer, 5′- CCTGCTTCACCACCTTCTTGA -3′.

### Luciferase Reporter Assay

C2C12 cells were seeded into 24-well plates the day before transfection. Control or Kindlin 2 siRNA was transfected into C2C12 cells with Lipofectamine RNAiMAX (Invitrogen). At 24 h post transfection, 100 ng of super 8x TOPFlash/FOPFlash plasmid with 1 ng of pRL were transfected per well using Lipofectamine 2000. After 24 h, C2C12 cells were induced myogenic differentiation for two days. The reporter activity was measured using a Dual-lusiferase Reporter Assay System (Promega).

### Co-immunoprecipitation (Co-IP)

A Co-IP assay was performed as described previously [14]. Briefly, total cell lysates were incubated with antibodies at 4°C for 4 h followed by incubating with protein A/G-Sepharose (Santa Cruz Biotechnology) overnight. After the beads were washed three times with NP40 buffer, the bound proteins were eluted with 2 × SDS loading buffer at 100°C for 5 min. The immunoprecipitates were analyzed by Western blotting with different antibodies.

### RNA Interference (RNAi)

Sequences of RNA interference (RNAi) oligonucleotides were as follows: Control siRNA, UUCUCCGAACGUGUCACGU; Kindlin 2 siRNA, AAGUUGG UGGAAAAACUCGAU.

### Immunofluorescence and Confocal Microscopy

After the cells were fixed with 4% paraformaldehyde solution at RT for 15 min, they were treated with 0.5% Triton X-100 at 37°C for 5 min and blocked with 5% BSA at room temperature for 1 h. The cells were then incubated with 1∶100 dilution of anti-Kindlin 2, anti-β-catenin, anti-active β-catenin or anti-TCF4 antibodies overnight at 4°C, and then with a 1∶100 dilution of Alexa Fluor 488 or 568-conjugated IgG (Invitrogen) for 1 h at room temperature. The images were captured with a TCS SP5 confocal microscope (Leica, Germany).

### Subcellular Fraction

Cells were rinsed twice in cold PBS, and then incubated with buffer A (50 mM Tris-HCl pH 7.8, 420 mM NaCl, 1 mM EDTA, 0.5% NP40, 0.34 M sucrose, 10% glycerol, 1 mM Na_3_VO_4_, and protease inhibitor mixture) for 5 min on ice. After the cells were scraped and centrifuged, the supernatant was the cytoplasmic fraction. Then the pellet was lysed in buffer B (10 mM HEPES, pH 7.9, 10 mM KCl, 1.5 mM MgCl_2_, 0.34 M sucrose, 10% glycerol, 0.1% Triton X-100, protease inhibitor mixture). After centrifuging, the supernatant was the nuclear fraction.

### Chromatin Immunoprecipitation (ChIP) Assay

A kit of Magna ChIP™ (Millipore, Catalog # 17-610) was used to perform chromatin immunoprecipitation (ChIP) assay. The antibodies used in ChIP assay are anti-β-catenin (BD), anti-Kindlin 2 (Millipore) and anti-TCF4 (Upstate). The primers for quantitative ChIP (q-ChIP) PCR were as follows: myogenin-F, GAATCACATGT AATCCACTGGA; myogenin-R, ACGCCAACTGCTGGGTGCCA.

## Results

### Kindlin 2 is Required for Muscle Cell Differentiation

**Figure 1:**
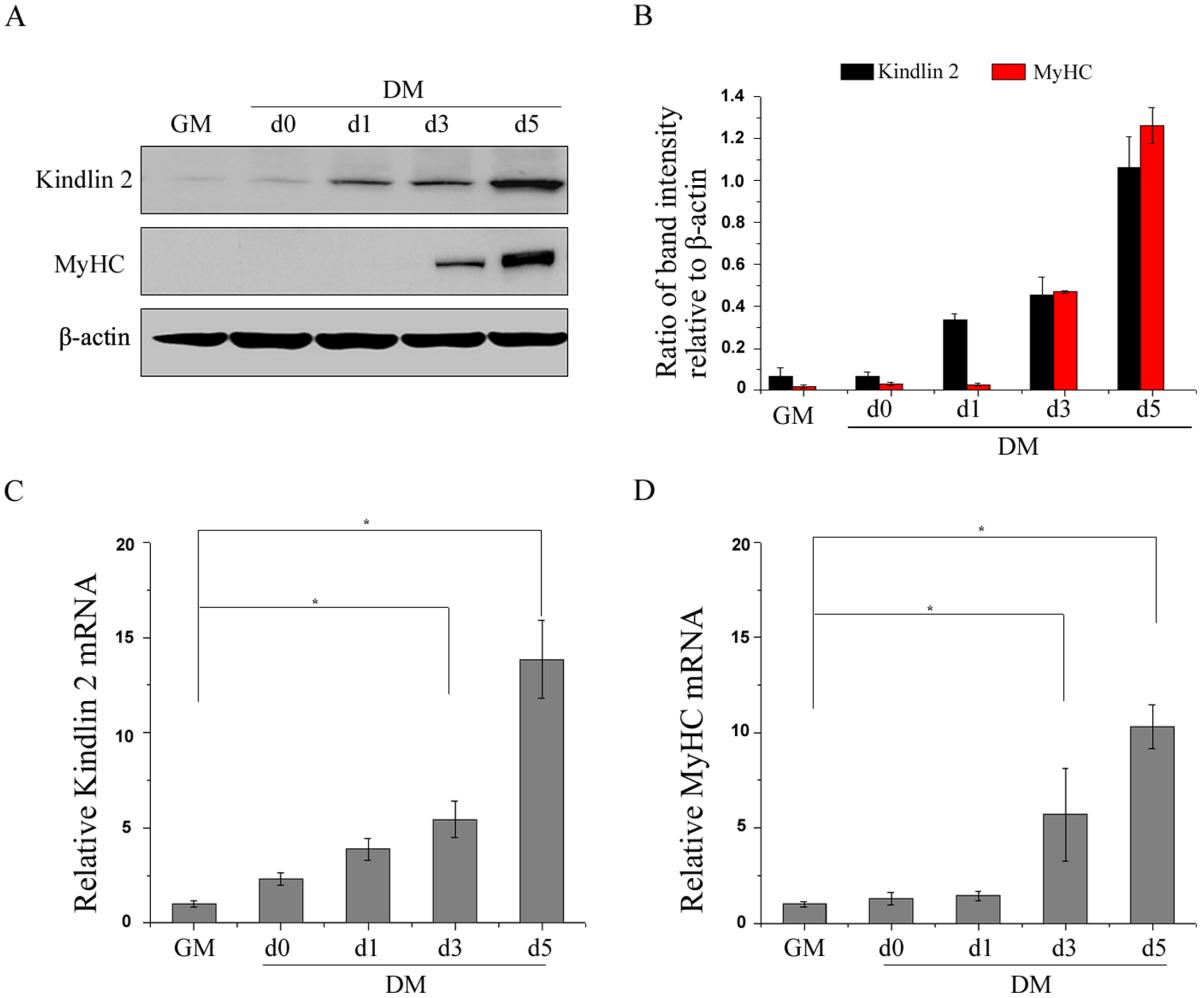
Kindlin 2 is activated during muscle cell differentiation. (A) C2C12 cells were cultured in growth medium (GM) or differentiation medium (DM) for 0, 1, 3, 5 days. Proteins were extracted from the cells at different stages, and Western blot (WB) assays were performed using the indicated antibodies. β-actin was used as a loading control. (B) Protein bands in A were scanned, and relative band intensities were normalized for each β-actin band. The column diagrams represent average relative band intensity with standard error from three independent experiments. (C–D) Total RNA was extracted from the cells at different stages. The mRNA levels of Kindlin 2 and MyHC were examined by qPCR. Error bars indicate s.d. values, n = 3; * indicates p<0.05 by Student’s *t*-test.

To mimic the process of muscle development *in vitro*, C2C12 murine myoblasts are used as a model of skeletal muscle differentiation. In C2C12 cell culture model system, C2C12 cell differentiation is triggered by serum removal. Proliferating C2C12 cells are cultured in growth medium and regarded as undifferentiated cells (GM stage). Upon serum removal, the proliferation of C2C12 cells is arrested, and the elongation and fusion of C2C12 cells are induced (DM stage). Eventually, multinucleated myotubes are formed. To evaluate the role of Kindlin 2 during skeletal muscle differentiation, we detected the levels of Kindlin 2 at different stages of cell differentiation, including proliferating C2C12 cells with 50% confluence (GM), proliferating C2C12 cells with 100% confluence (DM day 0) and the first five days after serum removal (DM day 1–5). As determined by Western blot analysis, the level of Kindlin 2 was low in undifferentiated cells (GM) (Fig. 1A–B). Upon C2C12 cell differentiation, the level of Kindlin 2 was elevated. At day 5 of cell differentiation, Kindlin 2 level was increased about 10 folds comparing with that in undifferentiated cells (Fig. 1B). Myosin heavy chain (MyHC) is a differentiated marker of muscle, which is induced at day 3 of cell differentiation (Fig. 1A), reflecting the beginning of myogenesis. Further, real-time PCR assays were performed and results showed that both Kindlin 2 and MyHC were increased in the mRNA levels during cell differentiation (Fig. 1C–D). Together, these data indicated that Kindlin 2 was activated during muscle cell differentiation in both protein and mRNA levels.

**Figure 2:**
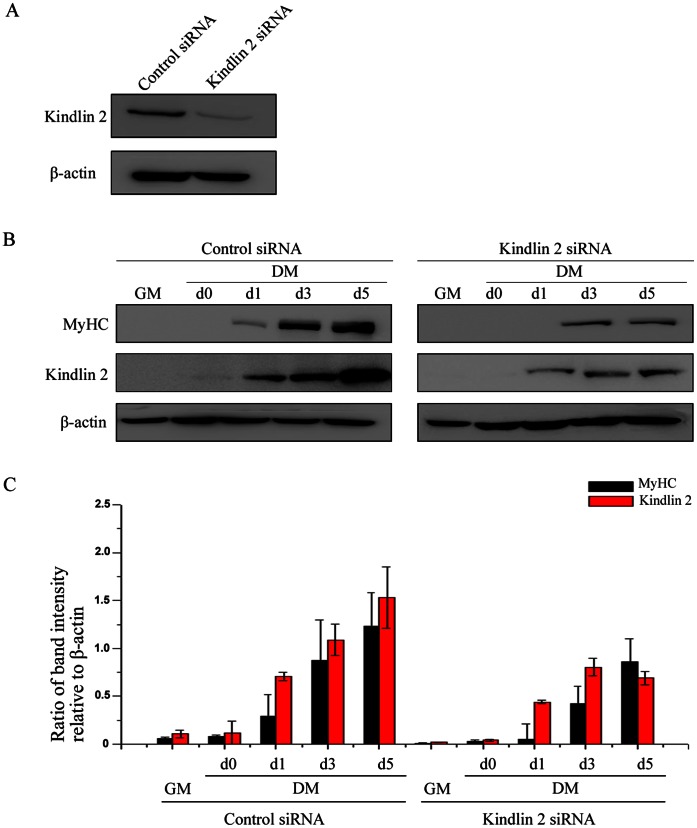
Knockdown of Kindlin 2 inhibits muscle cell differentiation. (A) The efficacy of Kindlin 2 siRNA was detected by Western blot. (B) Control or Kindlin 2 siRNA was transfected into C2C12 cells. After 24 hr, C2C12 cells were induced myogenic differentiation. At day 3 of differentiation, Western blot analysis was carried out with the indicated antibodies. (C) Protein bands in C were scanned, and relative band intensities were normalized for each β-actin band. The column diagrams represent average relative band intensity with standard error from three independent experiments.

Furthermore, to confirm whether Kindlin 2 is required for muscle cell differentiation, small interfering RNA (siRNA) to murine Kindlin 2 gene was designed and synthesized. The efficacy of siRNA was determined by Western blot in Fig. 2A. To explore the effect of Kindlin 2 knockdown on MyHC expression, Western blot analysis was performed and results showed that MyHC expression was inhibited in differentiated C2C12 cells with depleted Kindlin 2 (Fig. 2B–C). These data indicated that knockdown of Kindlin 2 led to an obvious delay in C2C12 cell differentiation, suggesting that Kindlin 2 is required for muscle cell differentiation.

### Kindlin 2 Activates Wnt Signaling in Myogenic Differentiation

**Figure 3:**
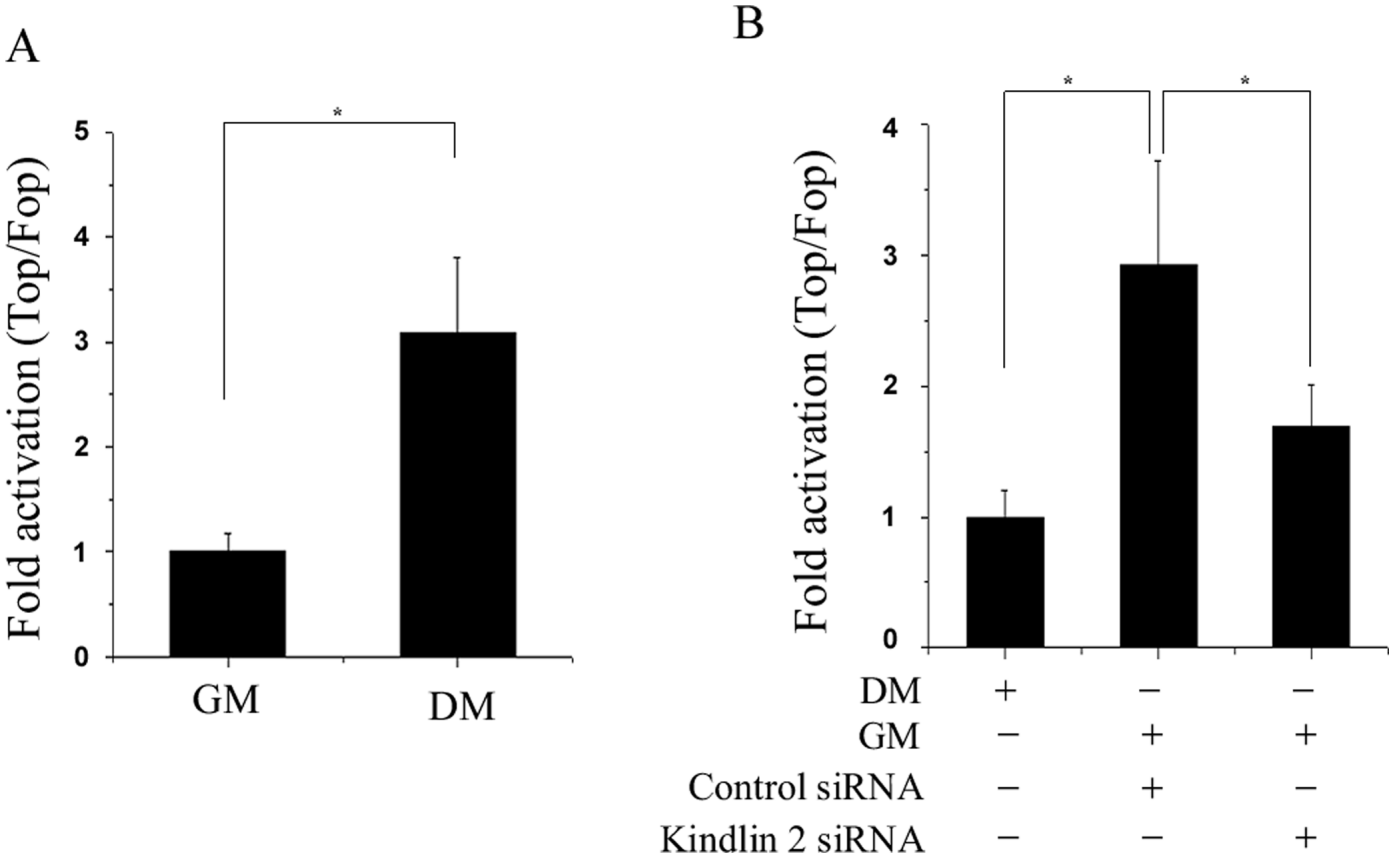
Kindlin 2 is required for activating Wnt signaling. (A) 100 ng Super8xTOPFlash/FOPFlash plasmid with 1 ng of pRL were transfected into C2C12 cells. After 24 hr, C2C12 cells were induced myogenic differentiation for two days. The luciferase reporter activity was measured in undifferentiated cells (GM) and differentiated cells (DM day 2). (B) SuperTop/Fopflash reporter assays were performed in GM cells and DM cells with or without Kindlin 2 knockdown. Error bars indicate S.D. values, n = 3; * indicates p<0.05 by Student’s *t*-test.

Given the critical role of Wnt signaling in muscle development, SuperTop/Fopflash reporter assay was performed. SuperTopflash is a luciferase reporter of β-catenin/TCF4-mediated transcriptional activation, which contains seven TCF/LEF binding sites. SuperFopflash is an appropriate control plasmid, which has some mutant TCF/LEF binding sites. As shown in Fig. 3A, β-catenin/TCF4-mediated transcription was significantly activated in differentated C2C12 cells, indicating that Wnt signaling is involved in the muscle cell differentiation. Since Kindlin 2 is required for muscle cell differentiation as demonstrated above, it is worthy to explore whether Kindlin 2 mediates the activation of β-catenin/TCF4-mediated transcription during myogenic differentiation. To this end, small interfering RNA was used to knock down endogenous Kindlin 2 in C2C12 cells. After control or Kindlin 2 siRNA was transfected into C2C12 cells for 24 hr, C2C12 cells were induced myogenic differentiation. At day 2 of cell differentiation, SuperTopflash reporter assays were carried out to compare the activation of β-catenin/TCF4-mediated transcription. Results showed that knockdown of Kindlin 2 partially abolished the activation of β-catenin/TCF4-mediated transcription induced by muscle cell differentiation (Fig. 3B). These data indicated that Kindlin 2 is required for the activation of Wnt signaling during myogenic differentiation.

**Figure 4:**
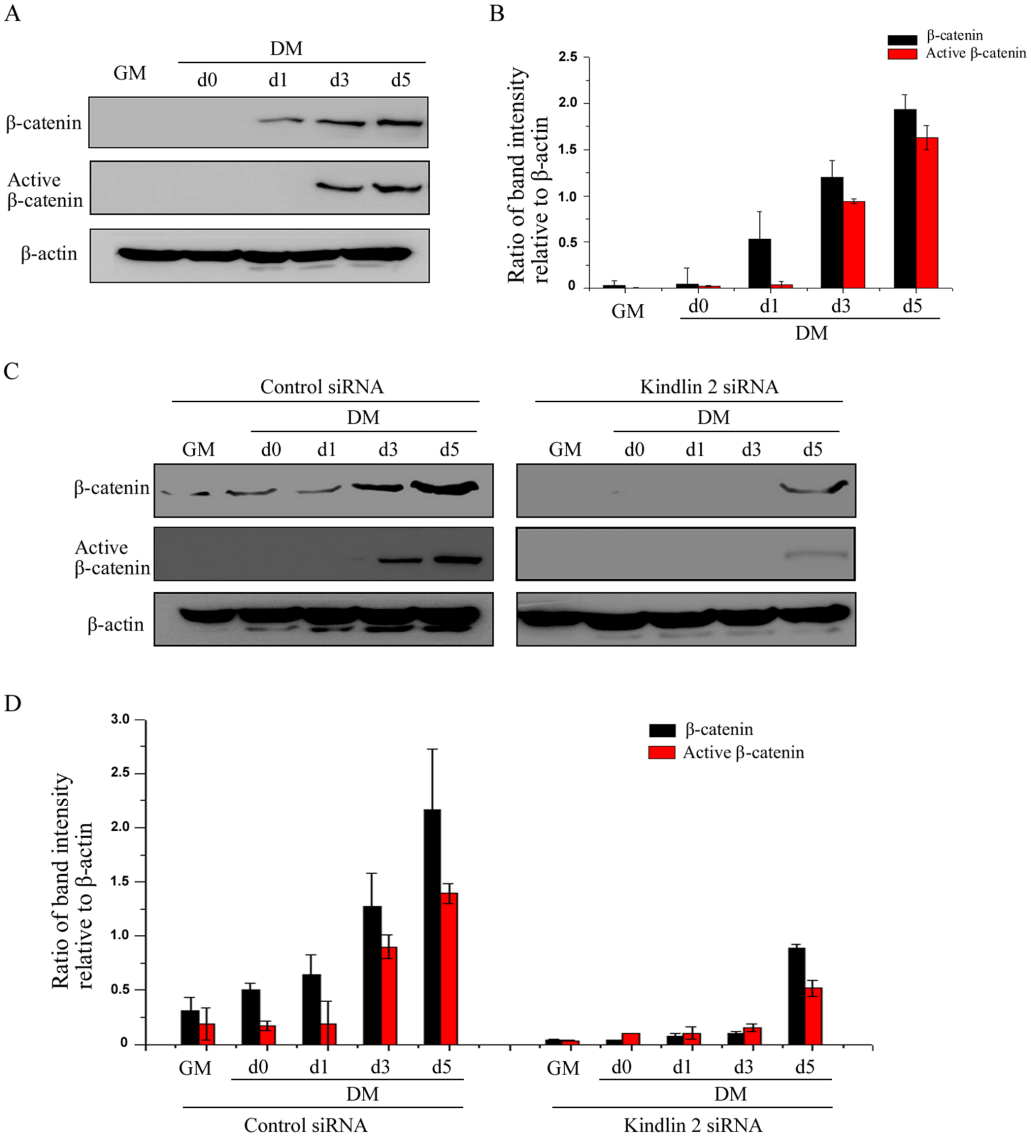
Kindlin 2 mediates the activation of β-catenin idifferentiation. (A) Proteins were extracted from the cells at different stages, and WB assays were performed using the indicated antibodies. (B) Protein bands in A were scanned and relative band intensities were normalized for each β-actin band. The column diagrams represent average relative band intensity with standard error from three independent experiments. (C) Control or Kindlin 2 siRNA was transfected into C2C12 cells. After 24 hr, C2C12 cells were induced myogenic differentiation for 3 days. WB analysis was carried out with the indicated antibodies. (D) Protein bands in C were scanned, and relative band intensities were normalized for each β-actin band. The column diagrams represent average relative band intensity with standard error from three independent experiments.

To scrutinize how Kindlin 2 regulates Wnt signaling during myogenic differentiation, we investigated the regulation of Kindlin 2 on β-catenin. Firstly, the expression of β-catenin was detected in undifferentiated and differentiated C2C12 cell. As determined by Western blot analysis, β-catenin expression was obviously induced during C2C12 cells differentiation (Fig. 4A). The level of β-catenin was increased to about 7 folds at day 5 of C2C12 cell differentiation, compared with undifferentiated cells (Fig. 4B). Although the accumulation of β-catenin is a clear hallmark of Wnt signaling activation, only unphosphorylated β-catenin is sufficient to activate the target genes. Intrinsically, unphosphorylated β-catenin is the active form of β-catenin, also called active β-catenin [15], [16]. As expected, consistent with total β-catenin, active β-catenin was also induced expression during C2C12 cell differentiation in Fig. 4A–B. Next, to determine the regulation of Kindlin 2 on β-catenin during myogenic differentiation, the changes in β-catenin expression were observed in control siRNA or Kindlin 2 siRNA-treated C2C12 cells. Results showed that knockdown of Kindlin 2 inhibited the expression of β-catenin, especially active β-catenin (Fig. 4C–D), suggesting that β-catenin mediated the regulation of Kindlin 2 on Wnt signaling.

### Kindlin 2 is Enriched in the Nucleus in Myogenic Differentiation

**Figure 5:**
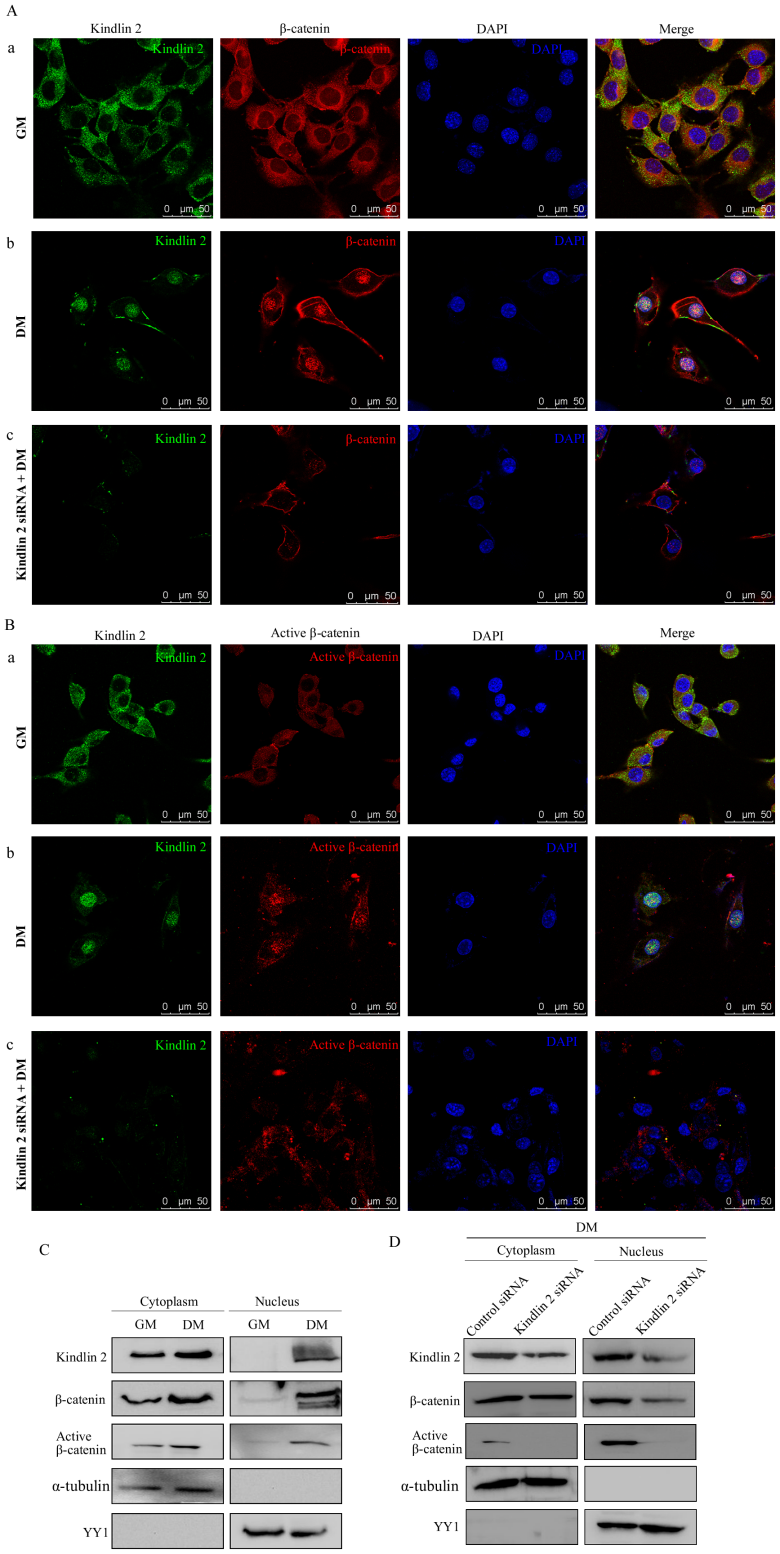
Kindin 2 is enriched in the nucleus in myogenic differentiation. (A) Immunofluorescence staining was performed in GM cells and DM cells (day 3) with or without Kindlin 2 knockdown using anti-Kindlin 2 (Alexa Flour 488, green) and anti-β-catenin (Alexa Flour 568, red) antibodies. Images were captured with a confocal microscopy. (B) Immunofluorescence staining for Kindlin 2 (Alexa Flour 488, green) and active β-catenin (Alexa Flour 568, red). (C) Both cytoplasmic and nuclear lysates were extracted from GM and DM cells (day 3) for WB analysis using the indicated antibodies. The absence of α-tubulin demonstrates that the fraction was from the nucleus. The absence of YY1 indicates that the fraction was from the cytoplasm. (D) Both cytoplasmic and nuclear lysates were extracted from DM cells with or without Kindlin 2 knockdown, and WB was performed.

To determine the subcellular localization of Kindlin 2 or β-catenin, immunofluorescence staining was performed using anti-Kindlin 2 or anti-β-catenin antibodies. In undifferentiated C2C12 cells, either Kindlin 2 or β-catenin mainly resided in the cytoplasm (Fig. 5A–a). Upon differentiation, nuclear translocation of both Kindlin 2 and β-catenin was observed. At day 3 of cell differentiation, we observed that Kindlin 2 and β-catenin were enriched in the nucleus (Fig. 5A–b). As Kindlin 2 contains a nuclear localization signal (NLS) domain, we speculated whether the translocation of β-catenin is related to Kindlin 2. To this end, we observed the alteration of β-catenin localization in Kindlin 2-depleted C2C12 cells compared with control cells. Results showed that, upon myogenic differentiation, depletion of Kindlin 2 abolished the nuclear accumulation of β-catenin (Fig. 5A–c), suggesting that Kindlin 2 may involve in the β-catenin translocation. Consistent with total β-catenin localization, active β-catenin was translocated into nucleus during C2C12 cell differentiation (Fig. 5B–a and 5B–b). When Kindlin 2 was knocked down, nuclear accumulation of active β-catenin was abolished (Fig. 5B–c).

Furthermore, distribution patterns of Kindlin 2 and β-catenin were determined by Western blot analysis of subcellular fraction. Results showed that, in undifferentiated cells, Kindlin 2, β-catenin or active β-catenin was only detected in the cytoplasmic fraction (Fig. 5C). Upon myogenic differentiation, the expression of Kindlin 2, β-catenin or active β-catenin in the nucleus was significantly increased (Fig. 5C). However, when Kindlin 2 was knocked down, the level of β-catenin or active β-catenin in the nucleus was markedly decreased compared with control cells (Fig. 5D). These results suggested that Kindlin 2 is involved in the nuclear translocation of β-catenin during myogenic differentiation.

### Kindlin 2 Forms a Tripartite Complex with β-catenin and TCF4

**Figure 6:**
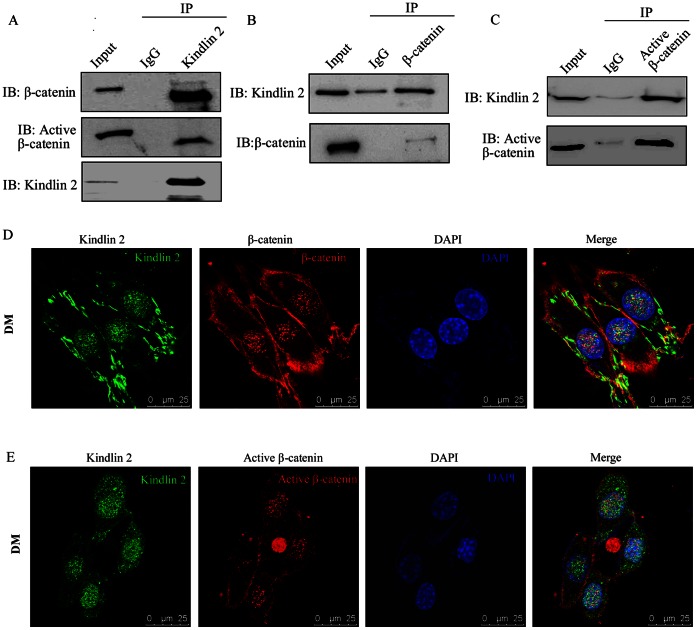
Kindlin 2 interacts with β-catenin. (A–C) Lysates from C2C12 cells (DM day 3) were extracted and then anti-Kindlin 2 (A), anti-β-catenin (B) or anti-active β-catenin (C) antibodies were used for Co-IP assays. (D–E) Immunofluorescence staining for Kindlin 2 (Alexa Flour 488, green) and β-catenin (D) or active β-catenin (E) (Alexa Flour 568, red) was performed in C2C12 cells (DM day 3). Images were captured with a confocal microscopy.

To investigate the physical association between Kindlin 2 and β-catenin *in vivo*, the interaction of Kindlin 2 and β-catenin was identified by co-immunoprecipitation (Co-IP) assay. As shown in Fig. 6A, endogenous β-catenin and active β-catenin were co-immunoprecipitated by agarose-conjugated anti-Kindlin 2 antibodies in cell lysates of differentiated C2C12 cells (Fig. 6A). Reciprocal co-immunoprecipitation assays were also performed with anti-β-catenin or anti-active β-catenin antibodies (Fig. 6B–C). These data demonstrated that Kindlin 2 forms a complex with β-catenin in differentiated C2C12 cells. To further explore where the two molecules bind together, immunofluorescence assay was performed and a clearly nuclear co-localization of Kindlin 2 and β-catenin or active β-catenin was observed in Fig. 6D–E.

**Figure 7:**
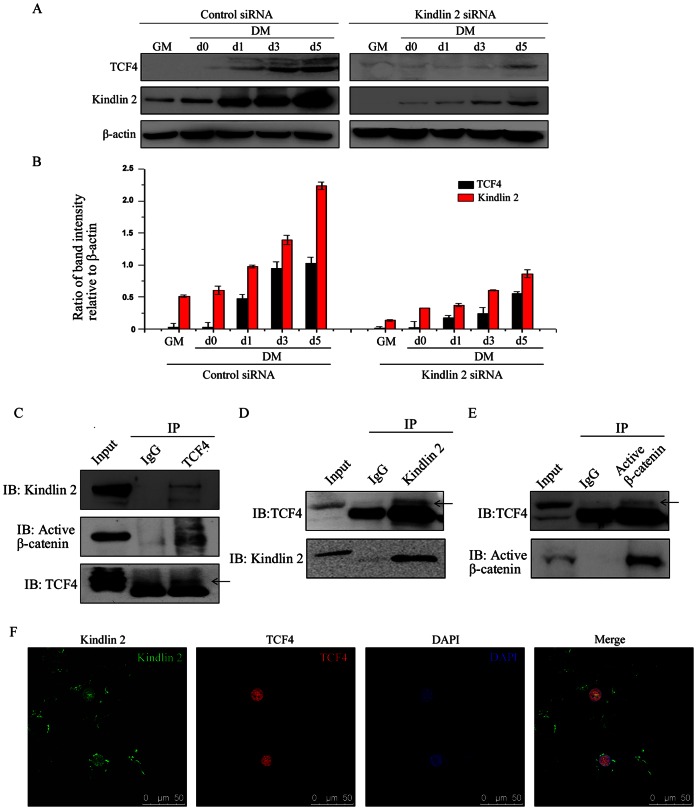
Kindlin 2 forms a complex with active β-catenin and TCF4. (A) Control or Kindlin 2 siRNA was transfected into C2C12 cells. After 24 hr, C2C12 cells were induced myogenic differentiation. At different time points, WB analysis was performed. (B) Protein bands in A were scanned, and relative band intensities were normalized for each β-actin band. The column diagrams represent average relative band intensity with standard error from three independent experiments. (C–E) Nuclear lysates from C2C12 cells (DM day 3) were prepared and then anti-TCF4 (C), anti-Kindlin 2 (D) or anti-active β-catenin (E) antibodies were used for Co-IP assays. (F) Immunofluorescence staining for Kindlin 2 (Alexa Flour 488, green) or TCF4 (Alexa Flour 568, red) was performed in C2C12 cells (DM day 3).

Nuclear β-catenin has been shown to interact with members of the TCF/LEF family to activate Wnt target gene expression. To uncover the relationship of Kindlin 2 and TCF/LEF family, we first explored the effect of Kindlin 2 on TCF4 expression. As determined by Western blot analysis, TCF4 was induced expression at day 1of C2C12 cell differentiation. When Kindlin 2 was knocked down, the induction of TCF4 expression was obviously delayed, suggesting that Kindlin 2 was involved in the regulation of TCF4 expression (Fig. 7A–B). To deeply explore the physical association of Kindlin 2, active β-catenin and TCF4, we extracted the nuclear protein of differentiated C2C12 cells, and reciprocal immunoprecipitation experiments were separately carried out with anti-Kindlin 2, anti-active β-catenin or anti-TCF4 antibodies. Results showed that the three molecules formed a tripartite complex (Fig. 7C–E). Moreover, the co-localization of Kindlin 2 and TCF4 in the nucleus was identified by immunofluorescence staining (Fig. 7F). These data demonstrated that the tripartite complex of Kindlin 2– active β-catenin – TCF4 was formed in the nucleus of differentiated C2C12 cells, suggesting that Kindlin 2 is involved in the TCF4-mediated Wnt target gene expression.

### Kindlin 2 Occupies the Promoter of Myogenin to Activate its Expression

**Figure 8:**
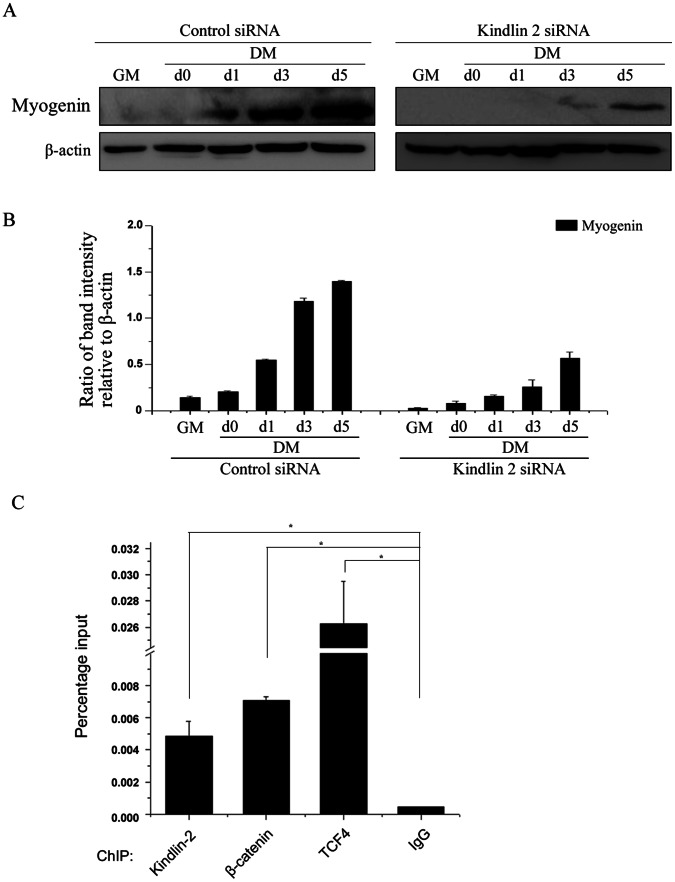
Kindlin 2 activates myogenin expression. (A) Control or Kindlin 2 siRNA was transfected into C2C12 cells. After 24 hr, C2C12 cells were induced myogenic differentiation. At different time points, WB analysis was performed. (B) Protein bands in A were scanned, and relative band intensities were normalized for each β-actin band. The column diagrams represent average relative band intensity with standard error from three independent experiments. (C) C2C12 cells were induced myogenic differentiation for five days. ChIP assay was performed for detecting the occupancy of the tripartite complex at myogenin promoter. The indicated antibodies were used to perform ChIP assays. To quantify the ChIP-enriched DNA, qPCR was carried out. Each data point is the average of duplicates from a representative experiment. Experiments were repeated three times. * indicates p<0.05 by Student’s *t*-test.

Myogenic regulatory factors function to induce the expression of some muscle specific proteins, such as, alpha-actin, myosin heavy and light chains, tropomyosin, troponin-C and troponin-I, which are critical for muscle development. Myogenin, a typical myogenic regulatory factor, was obviously activated during muscle cell differentiation (Fig. 8A–B). Further, knockdown of Kindlin 2 delayed the expression of myogenin (Fig. 8A–B), indicating that Kindlin 2 plays an important role in regulating the expression of myogenin. Moreover, to clarify the direct regulation of Kindlin 2 on myogenin, Chromatin Immunoprecipitation (ChIP) assay was carried out and results indicated that the tripartite complex of Kindlin 2, β-catenin and TCF4 co-occupied on the promoter of myogenin (Fig. 8C). These data demonstrated that Kindlin 2 does activate myogenin expression during muscle cell differentiation, suggesting that Kindlin 2 plays a critical role in muscle development.

## Discussion

Most studies of Kindlin 2 focused on its binding to integrin at focal contacts [9], [10]. Kindlin 2 has been confirmed to be required for the muscle development in an integrin-binding dependent manner [13]. Dowling et al. found that the function of Kindlin 2 in regulating cell migration and adhesion is critical during myoblast fusion and myocyte elongation. Our previous studies have indicated that the Kindlin 2 located in the nucleus plays an important role in regulating gene expression, which is independent on the binding of integrin [14], [17]. In this study, we found that Kindlin 2 binds β-catenin together in the nucleus, promoting the transcription of myogenic regulatory factor myogenin. Taken these studies together, Kindlin 2 has multiple important roles in the regulation of muscle development. On one hand, Kindlin 2 enters into nucleus and activates Wnt signaling, enhancing the expression of myogenic regulatory factor myogenin. On the other hand, Kindlin 2 is enriched at focal contacts to promote myoblast migration and adhesion.

Although it is well established that the nuclear translocation of β-catenin is a key factor of Wnt signaling activation, the detail mechanisms remain elusive. Smad3 is reported to enhance the stability of β-catenin and facilitate the nuclear translocation of β-catenin in chondrocytes [18]. In 2011, Zhang et al. found that FoxM1 is important for promoting β-catenin nuclear localization in glioma [19]. In this study, we found that both Kindlin 2 and β-catenin were translocated into nucleus during C2C12 cell differentiation. However, when Kindlin 2 was knocked down, the nuclear β-catenin was markedly decreased (Fig. 5). Moreover, Kindlin 2 was found containing a nuclear localization signal (NLS) [11], suggesting that Kindlin 2 may play a direct role in promoting the translocation of β-catenin during muscle cell differentiation, which leaves an opportunity for future investigation.

Kindlin 2– β-catenin complex was originally confirmed in breast cancer cells, in which Kindlin 2 was recruited by β-catenin to activate Wnt target gene AXIN2, and, in turn, promote the EMT process [14]. Here, we identified that the regulation of Kindlin 2 on Wnt signaling is required for the skeletal muscle differentiation. Kindlin 2 forms a tripartite complex with active β-catenin and TCF4 and co-occupies on the promoter of myogenin to activate myogenin expression (Fig. 7–8). Thus, disruption of the tripartite complex inhibits myogenin expression, which may lead to the delay of muscle differentiation.
